# Clinical epidemiology for comprehensive kidney care: a framework for developing clinical research questions, from biomarkers to patient-reported outcomes

**DOI:** 10.1007/s10157-026-02822-z

**Published:** 2026-01-31

**Authors:** Noriaki Kurita

**Affiliations:** 1 Department of Clinical Epidemiology, Graduate School of Medicine, Fukushima Medical University, 1 Hikarigaoka, Fukushima, Fukushima Japan; 2 Department of Innovative Research and Education for Clinicians and Trainees (DiRECT), Fukushima Medical University Hospital, Fukushima, Fukushima Japan; 3 Division of Rheumatology, Department of Medicine, Showa Medical University, Shinagawa-Ku, Tokyo, Japan

**Keywords:** Clinical epidemiology, Research question framework, Patient-reported outcomes, Biomarker evaluation, Physician–patient communication, Descriptive studies

## Abstract

Clinical nephrology research is increasingly challenged by the need to translate complex patient experiences, emerging biomarkers and treatments, and an expanding methodological tools into improvements in care. Clinical epidemiology provides a bridge between bedside questions and population science; however, its role is often narrowly perceived as clinical statistics rather than as a discipline centered on research conceptualization and design. In this invited review, I reflect on how clinical questions arising from routine nephrology practice can be systematically developed into clinically relevant research through a nephrologist–epidemiologist’s lens. First, drawing on our experience and illustrative examples, I describe how rethinking care processes through established frameworks—such as the structure–process–outcome model—can support clinicians in formulating answerable questions that matter to patients. Second, I expand the lens beyond traditional nephrology to incorporate perspectives from social medicine, emphasizing trust, hope, and patient-reported outcomes as integral components of chronic kidney disease care. Finally, this review highlights how clinical questions can be embedded within clinical research design frameworks to clarify research objectives across diagnosis, treatment, prognosis, and etiology. In an era of rapid methodological diversification, the nephrologist–epidemiologist’s unique contribution may lie in cultivating a sharpened lens: the ability to discern relevant clinical questions and sustain deep clinical reasoning. By doing so, clinical epidemiology can continue to guide research that advances comprehensive and patient-centered kidney care.

## From the hospital to epidemiology: how a clinical question shaped my research path

I would like to begin by sharing a clinical question that emerged early in my career as a nephrologist. I was struck by the severity of aortic stenosis in patients undergoing hemodialysis, a condition that often caused hemodynamic instability during dialysis sessions.

While observing surgically excised aortic valves under a microscope in the pathology department, I noticed not only extensive calcification but also deposits suggestive of amyloid. These specimens were positive for both Congo red staining and β2-microglobulin, a finding I encountered repeatedly. From these observations, I hypothesized that dialysis-related amyloid deposition might partly contribute to the development of severe aortic stenosis in this population.

I wanted to transform this clinical observation into scientific evidence. By chance, I attended a 1-week clinical research summer camp led by Professor Shunichi Fukuhara at Kyoto University, where a single slide left a lasting impression: clinical research educators will be essential in the future. Motivated by this message, I pursued formal training at the School of Public Health at Kyoto University to address my clinical question. This effort ultimately resulted in a published study, [1] marking the starting point of my journey into epidemiology grounded in clinical practice.

## Supporting clinicians by rethinking care processes

**Fig. 1:**
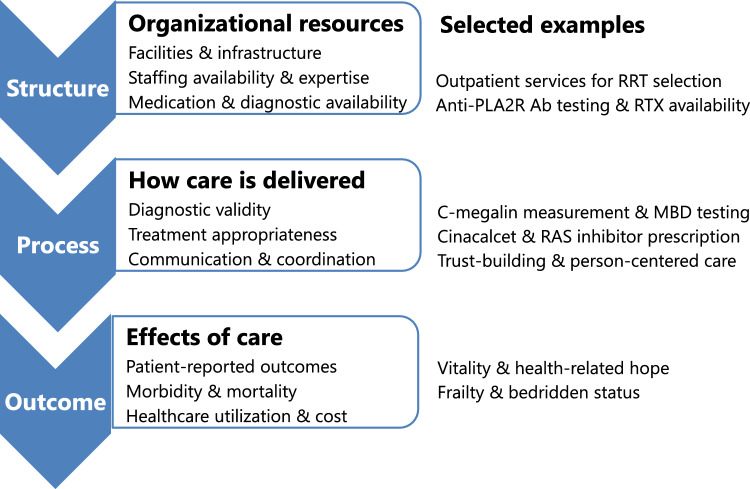
Examples of nephrology research mapped to the structure–process–outcome framework. RRT: Renal Replacement Therapy; PLA2R: Phospholipase A2 Receptor; Ab: Antibody; RTX: Rituximab; MBD: Mineral and Bone Disorder; RAS: Renin–Angiotensin System

The Donabedian model, proposed in 1980, conceptualizes healthcare quality across three domains: structure, process, and outcome (Fig. 1). [2] *Structure* refers to the settings and resources in which care is delivered. *Process* represents what is done in delivering care—including diagnostics, treatments, and communication. *Outcomes* refer to the resulting states of patients and health systems. [3]

This model has long served as the basis for quality improvement research and practice [2] and is frequently referenced in the nephrology literature. [4]

It was also a core concept in my postgraduate training at the School of Public Health, Kyoto University. Guided by this model, I have consistently sought to design clinical research aimed at improving one or more elements of this structure–process–outcome continuum. I believe that indicators situated within this framework represent potentially modifiable targets for clinicians and researchers seeking to improve nephrology care through clinical research.

The following sections introduce outcome-oriented studies—spanning treatment appropriateness, diagnostic validity, patient-reported outcomes (PROs), and morbidity—conducted from the perspective of a practicing nephrologist.

## Treatment appropriateness: cinacalcet and RAS inhibitors

### Cinacalcet

One of the most challenging questions I encountered as both a nephrologist and a clinical researcher concerned the effectiveness of cinacalcet in real-world practice. The landmark EVOLVE trial reported no reduction in all-cause mortality or cardiovascular events among hemodialysis patients with secondary hyperparathyroidism treated with cinacalcet. [5] However, this trial enrolled only patients with intact parathyroid hormone (iPTH) levels ≥ 300 pg/mL. This criterion differed substantially from Japanese clinical practice, where many patients are managed at iPTH levels below 300 pg/mL in accordance with national guidelines. This discrepancy led to a key clinical question: does the effectiveness of cinacalcet vary according to baseline iPTH levels?

Using observational data from the MBD-5D Study— a large multicenter cohort led by Drs. Shunichi Fukuhara, Tadao Akizawa, and Masafumi Fukagawa that enrolled patients with secondary hyperparathyroidism undergoing hemodialysis from more than 80 facilities across Japan—we found that the association between cinacalcet initiation and reduced all-cause mortality became stronger with increasing baseline iPTH levels. Specifically, no mortality reduction was observed among patients with iPTH < 300 pg/mL (incidence rate ratio [IRR], 1.07; 95% confidence interval [CI] 0.77–1.48), whereas progressively lower mortality was observed among those with iPTH 300– < 500 pg/mL (IRR, 0.88; 95% CI 0.61–1.29) and ≥ 500 pg/mL (IRR, 0.49; 95% CI 0.29–0.82). [6]

Methodologically, this study addressed the challenge of evaluating treatment effectiveness in an observational setting with time-varying exposures and confounders. Because patients contributed repeated laboratory values, prescriptions, and outcomes over time, we applied marginal structural models to appropriately account for time-dependent confounding. [7] This approach allowed us to estimate how outcomes would differ if patients in this population were treated with cinacalcet compared with a counterfactual scenario in which they were not treated.

### RAS inhibitors

Building on this experience, we applied the same methodology to a different clinical context. As part of the Nephrotic Syndrome Working Group of the guideline development subcommittee of the Investigative Research on Refractory Kidney Disorders program, we accessed data from the Japanese Nephrotic Syndrome Cohort Study. In this cohort, renin–angiotensin system (RAS) inhibitors were newly prescribed at various time points after diagnosis and initiation of immunosuppressive therapy, constituting a time-varying exposure. [8] Applying a marginal structural model, we examined whether initiation of RAS inhibitors was associated with complete remission among patients with primary nephrotic syndrome receiving immunosuppressive therapy. [9]

## Diagnostic validity: urinary C-megalin and MBD marker testing

### Urinary C-megalin

Diabetic kidney disease (DKD) has traditionally been characterized by a progression from hyperfiltration to microalbuminuria, macroalbuminuria, and subsequent decline in kidney function. However, recognition of non-proteinuric DKD has highlighted the limitations of relying solely on urinary albumin excretion to detect early kidney injury. [10] Although urinary albumin remains a key predictor of kidney failure, there is a strong clinical need for novel urinary biomarkers that rise earlier in the disease course.

Urinary C-megalin has emerged as a promising candidate biomarker. In collaboration with Dr. Yasuaki Hayashino, we investigated whether urinary C-megalin could identify the development of persistent microalbuminuria at an earlier stage. Urinary C-megalin was associated with incident microalbuminuria independently of urinary albumin levels, with the association being most pronounced among individuals with lower baseline albumin excretion. [11]

Because the value of a novel diagnostic marker depends on whether it provides information beyond existing biomarkers, [10] we assessed its incremental performance. Adding urinary C-megalin improved discrimination for predicting microalbuminuria onset. [11] Extending this work, we further demonstrated that, particularly among DKD patients who had not yet progressed to macroalbuminuria, urinary C-megalin was independently associated with subsequent kidney function decline after adjustment for baseline urinary albumin and estimated glomerular filtration rate. [12]

### MBD marker testing

Questions regarding the appropriate testing frequency of mineral and bone disorder (MBD) markers in patients receiving hemodialysis can also be framed as issues of diagnostic validity. The aforementioned MBD-5D study demonstrated substantial inter-facility variation in testing practices, with a notable proportion of facilities performed testing more frequently than guideline recommendations: 4.8% measured serum calcium and phosphorus levels weekly, and 27.5% measured parathyroid hormone (PTH) every 1–2 months. [13]

Hypothesizing that this variation reflected attempts to more intensively manage biochemical abnormalities, we conducted further analyses using data from the same MBD-5D study. Among patients whose values exceeded guideline-specified target ranges, more frequent monitoring was associated with a higher likelihood of subsequent target attainment. In particular, weekly monitoring of serum calcium and monthly measurement of PTH were associated with improved attainment of guideline-recommended ranges at follow-up. [14]

## Patient-reported outcomes: vitality and health-related hope

### Vitality

PROs capture dimensions of health that are often more important and relevant to patients than clinician-assessed measures. When patient-important PROs are not incorporated into clinical research, evidence gaps may arise that hinder shared decision-making (SDM). [15] Reflecting this concern, the Standardized Outcomes in Nephrology initiative has identified fatigue as a core outcome in hemodialysis trials. [15] Fatigue encompasses subjective tiredness, lack of energy, and mental exhaustion, and represents one of the most burdensome symptoms experienced by patients undergoing hemodialysis.

In this context, we focused on vitality—the conceptual opposite of fatigue—using a single-item energy question from the SF-36 vitality domain (“Did you have a lot of energy?”). [16] Higher self-reported energy was inversely associated with cardiovascular hospitalization and mortality among patients receiving hemodialysis. Patients reporting energy “all of the time” experienced a 37% lower rate of cardiovascular events and death compared with those reporting “none of the time.”

Vitality was also associated with objective physiological parameters. Higher energy levels correlated with higher body mass index, higher serum albumin, and greater dialysis dose, whereas lower energy levels were associated with hypnotic or antidepressant use and tachycardia. [16] Although causal mechanisms cannot be inferred, these findings suggest that vitality is closely linked to nutritional status, dialysis adequacy, and medication burden.

Taken together, these observations led us to conceptualize vitality as a “vital sign” of quality of life—one that can be measured simply and repeatedly and may offer clinicians a practical indicator to support comprehensive kidney care.

### Health-related hope

PROs can also visualize and quantify the burden that disease imposes on patients and society (Table 1). By making this burden explicit, PROs help clinicians recognize unmet needs and identify opportunities to improve care delivery. As dialysis has become a long-term life-sustaining therapy—allowing some patients in Japan to survive for nearly half a century [17]—clinicians are increasingly challenged to support not only survival but also patients’ values and life priorities.

**Table 1:** Applications of patient-reported outcomes in clinical research and practice

Objectives in clinical research and practice	Examples
Outcomes in studies evaluating treatment effectiveness	Differences in physical and mental QOL according to modes of transportation to outpatient hemodialysis facilities [45]
Making clinicians aware of disease- and symptom-related burden on patients and society	Variation in levels of HR-Hope across different stages and severities of CKD [20]
Predictors of future clinical outcomes	Associations between a single-item self-reported vitality question and subsequent cardiovascular hospitalization and mortality [16]
Factors explaining treatment adherence and treatment resistance	Associations of HR-Hope with medication adherence, [22] objective indicators of self-management, [20] and future distress related to fluid and dietary restrictions [21]
Use in routine clinical practice at the individual patient level	Longitudinal monitoring of symptoms and psychological status using validated scales (e.g., ESAS-r: Renal) [46]

*QOL* quality of life; *CKD* chronic kidney disease; *HR-Hope* Health-Related Hope; *ESAS-r: Rena*l Edmonton Symptom Assessment System–revised: Renal

In this context, hope may serve as an internal resource that enables patients to live in accordance with their values. Indeed, Dr. Jerome Groopman, former Professor at Harvard Medical School, explored the meaning of hope through his experiences with patients with cancer, colleagues, and his own illness. In *The Anatomy of Hope*, he described “realistic hope”—not as naïve optimism or guaranteed cure, but as a source of meaning and strength that enables patients to endure uncertainty and treatment. [18]

Motivated by this perspective, we developed the Health-Related Hope Scale (HR-Hope), an 18-item scale assessing patients’ future-oriented values across three domains—health and illness, roles and connectedness, and something to live for (ikigai)—with the support of Drs. Shunichi Fukuhara, Takafumi Wakita, and Yugo Shibagaki. [19] HR-Hope scores were lowest among patients with stage 5 chronic kidney disease (CKD) before dialysis initiation, compared with those with stage 4 CKD or those already receiving dialysis. [20] This pattern suggests that physical limitations due to uremic symptoms and anxiety related to impending renal replacement therapy (RRT) may contribute to reduced hope.

In addition, higher HR-Hope scores were independently associated with less subsequent distress related to fluid and dietary restrictions, even after adjustment for depressive symptoms. [21] These findings suggest that assessing HR-Hope may help clinicians identify patients experiencing uncertainty or hopelessness and provide opportunities to support SDM. Moreover, maintaining HR-Hope may help buffer the difficulties associated with long-term self-management and medication adherence. [22]

## Morbidity and mortality: frailty and bedridden status

As described above, an increasing number of patients are now surviving on dialysis for nearly half a century. This raises an important question: to what extent can patients receiving long-term dialysis maintain physical health compared with individuals not undergoing dialysis?

Using data from the Japanese Renal Data Registry, we examined the association between dialysis vintage and functional status in collaboration with Dr. Suguru Yamamoto. [17] Compared with patients receiving dialysis for less than 5 years, those with a dialysis duration of 30 years or longer had a 1.67-fold higher risk of frailty and a 1.66-fold higher risk of being bedridden, indicating a progressive increase in risk with longer dialysis therapy.

Although cross-sectional, these findings suggest a cumulative uremic burden associated with prolonged dialysis exposure and highlight the need to elucidate the mechanisms underlying dialysis-related complications and to improve dialysis technologies aimed at preventing functional decline. In this context, HR-Hope may serve as an important inner resource again, [47] as it may sometimes be the only remaining option for patients when substantial physical impairment severely limits other forms of action. [23, 24]

## Beyond the nephrologist: a social medicine and clinical epidemiology perspective

Research in clinical nephrology has traditionally focused on healthcare provider–level and patient–level factors, aiming to improve treatments, diagnostics, and patient outcomes. While this perspective remains essential, it captures only part of the broader landscape of care quality.

As framed earlier using the Donabedian model, optimizing care also requires attention to system-level factors (i.e., structure) and to the nature of healthcare provider–patient interactions. These dimensions—integral to social medicine and clinical epidemiology—help elucidate how care is delivered and experienced, and can influence outcomes for patients with CKD. Here, we present examples of nephrology research informed by this broader perspective.

## Staffing availability and expertise in outpatient RRT decision-making

Selecting RRT requires patients with CKD to consider not only their medical condition but also what matters most in their lives. SDM, which integrates clinical expertise with patients’ lived experiences, has been associated with value-concordant modality choice, less decisional regret, and better outcomes.

In Japan, a system-level incentive to support SDM in RRT selection was introduced in 2020 through a reimbursement add-on (*Jin-daitai-ryōhō shidō kanri-ryō*). This policy allows reimbursement for up to two structured outpatient sessions of at least 30 min, delivered collaboratively by physicians and nurses. Reimbursement eligibility depends on facility-level resources, including experienced full-time nephrologists and nursing staff with expertise in kidney care. However, many patients undergo RRT decision-making in settings that do not meet these criteria, leaving the real-world impact of this policy unclear.

To address this issue, led by Dr. Yugo Shibagaki, we examined system-level factors associated with patients’ retrospective perception of SDM during RRT selection across 49 facilities. We evaluated two factors: the availability of specialized outpatient services for RRT selection linked to reimbursement policy, and the number of outpatient visits dedicated to RRT selection, regardless of reimbursement eligibility. The latter required nursing involvement and a minimum consultation time of 30 min but did not depend on formal facility requirements. [25]

We found that the availability of specialized outpatient visits was not associated with increased perceived SDM (relative risk [RR] 1.17; 95% CI 0.76–1.82). In contrast, two or more outpatient visits for RRT selection were associated with a higher likelihood of perceived SDM (RR 1.59; 95% CI 1.05–2.42). [25] These findings suggest that reimbursement policies intended to promote SDM may be more effective if they support feasible, iterative outpatient engagement, particularly in resource-constrained settings.

## Medication and diagnostic availability: anti-PLA2R antibody testing and rituximab use

The KDIGO 2021 Clinical Practice Guideline states that positivity for serum anti-phospholipase A2 receptor (PLA2R) antibodies is sufficient to diagnose primary membranous nephropathy (pMN). In Japan, however, anti-PLA2R antibody testing is not covered by public health insurance, and the Evidence-Based Clinical Practice Guideline for Nephrotic Syndrome 2020 therefore suggests its use only when kidney biopsy is not feasible. [26]

Within this context, and again as part of the Nephrotic Syndrome Working Group of the guideline development subcommittee within the Investigative Research on Refractory Kidney Disorders program, we conducted a nationwide survey led by Dr. Takehiko Wada, involving more than 400 board-certified nephrologists from over 300 facilities. [27] Notably, 41.2% reported that they would not measure anti-PLA2R antibodies even when kidney biopsy was not feasible in patients with suspected pMN. The most commonly cited reason was the lack of insurance reimbursement and difficulty covering testing costs (63.7%).

Only 32.8% of physicians reported experience with anti-PLA2R antibody testing at their current institution. [28] Such experience was more common in university hospitals, high–kidney-biopsy–volume centers, and among physicians with longer clinical experience (≥ 21 years). Testing was most often supported by departmental research support (62.9%), followed by hospital budgets (17.9%), while direct patient payment accounted for 6.4%. These findings suggest that the absence of insurance coverage contributes to both financial barriers and institutional disparities in access to guideline-recommended diagnostics.

We applied the same survey framework to therapeutic decision-making, focusing on rituximab use in pMN. Rituximab is more effective and better tolerated than conservative therapy or cyclosporine monotherapy in pMN, and is recommended by KDIGO 2021 as a standard treatment option for idiopathic membranous nephropathy. In Japan, however, insurance coverage for rituximab is restricted to frequently relapsing or steroid-dependent nephrotic syndrome, leading the 2020 Japanese guideline to provide only a descriptive statement regarding its use in nephrotic pMN. [26]

Consistent with this restriction, only 21.8% of physicians reported experience using rituximab for pMN. [29] Although most rituximab treatments were covered by patients’ insurance (67.5%), a substantial proportion were paid by hospitals (12.0%) or directly by patients (7.2%). Notably, when rituximab was considered clinically beneficial, 48.4% of physicians reported refraining from its use, most commonly because of lack of coverage and the associated financial burden (79.3%).

Collectively, these findings indicate that limited insurance reimbursement is a major system-level barrier to the use of both anti-PLA2R antibody testing and rituximab in pMN management in Japan. Addressing these system-level constraints may be essential to ensure equitable access to evidence-based diagnostic and therapeutic care.

## Communication and coordination: trust-building and person-centered care

**Fig. 2:**
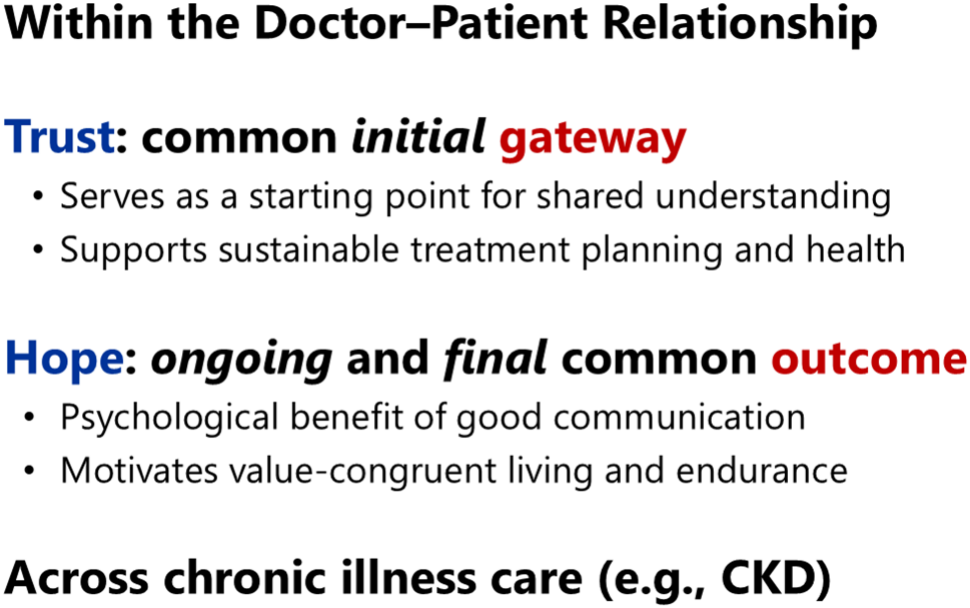
Trust and hope within the doctor–patient relationship across chronic illness care. CKD: chronic kidney disease

High-quality healthcare provider–patient interactions contribute to health outcomes both directly and indirectly. [30] In the care of chronic illness, **trust** can be conceptualized as a *common initial gateway*—a universal starting point that enables effective care (Fig. 2). When trust is established, it facilitates patients’ understanding of their illness, enables sustainable treatment planning, and contributes to long-term health maintenance. Paired with trust, **hope** may be viewed as an *ongoing and final common outcome* across chronic illness care. Hope motivates patients to live in accordance with their values and provides psychological strength to accept and endure illness and treatment over time.

To examine the role of trust in nephrology, we conducted a multicenter cross-sectional study investigating its contribution to medication adherence difficulties among patients receiving hemodialysis. Although higher health literacy is associated with fewer adherence difficulties, we demonstrated that trust in physicians partially mediates this relationship. [31] Specifically, patients with higher basic health literacy tended to report greater trust, which was associated with fewer adherence difficulties, likely through improved understanding of their condition and physicians’ medication explanations. Health literacy also exerted a direct effect independent of trust, presumably by improving patients’ comprehension of medication regimens and schedules.

Using data from the same study, we further examined the roles of person-centered care and trust. [32] Higher quality person-centered care was associated with greater trust in physicians. Such care includes effective care coordination and longitudinal relationships, encompassing attention to what matters most to patients and treating them as whole persons. Patients experiencing this type of care were more likely to develop trust in physicians, which in turn was associated with fewer medication-related difficulties. Moreover, person-centered care itself appeared to reduce adherence difficulties independently of trust.

Taken together, these findings provide quantitative support for the long-standing narrative that trust-building and person-centered care play an important role in sustaining medication adherence in CKD. They also illustrate that the art of medicine—communication, relationships, and empathy—and the evidence base of clinical care are inseparable in nephrology care.

## Embedding clinical questions within clinical research design frameworks

Classifying clinical questions by both their focus and research objective enables clinicians to select research designs that are well matched to the problems they seek to address (Table 2).

**Table 2:** A framework for designing clinical research across the focus of clinical questions and research objectives

	Focus of the clinical question			
	Diagnosis	Treatment	Prognosis	Etiology
Research objective				
Describing disease characteristics or clinical practice	★ Anti-PLA2R Ab—descriptive [27, 28]	★ Rituximab—descriptive [29]	★ AVF creation timing and natural history [36]	★
Evaluating diagnostic performance	★★★			
Examining associations between exposures and outcomes	MBD testing—guideline target achievement [14]	★★ Cinacalcet—MBD-5D study [6]	★★★ Dialysis duration and frailty [17]	★★★ Trust, hope, and adherence [20, 21, 31, 32]
Assessing treatment effectiveness		★★★ Cinacalcet—EVOLVE trial		
Predicting disease onset or prognosis	★★ C-megalin—microalbuminuria [11]		★★ REC^2^AP score [38]	

★ Descriptive clinical research

★★ Typical clinical research

★★★ Typical and ideal clinical research

The symbols (★, ★★, ★★★) represent increasing degrees of conceptual alignment between the clinical question and the research objective, rather than methodological superiority or inferiority.

Adapted from a Japanese-language review article authored by the present author. [34]

*Ab*: Antibody; *Anti-PLA2R Ab*: Anti–phospholipase A2 receptor antibody; *AVF*: Arteriovenous fistula; *CKD*: Chronic kidney disease; *EBM*: Evidence-based medicine; *EVOLVE trial*: Evaluation of Cinacalcet Therapy to Lower Cardiovascular Events trial; *GL*: Guideline; *iPTH*: Intact parathyroid hormone; *MBD*: Mineral and bone disorder; *MBD-5D Study*: Mineral and Bone Disorder Outcomes Study for Japanese CKD Stage 5D Patients; *RAS*: Renin–angiotensin system; *REC*^*2*^*AP score*: Renal function, Erythropoiesis-stimulating agent use, Charlson Comorbidity Index, Calcium, Albumin, Performance Status score

In evidence-based medicine , clinical questions are commonly categorized into four domains—diagnosis, treatment, prognosis, and etiology—while research objectives can be grouped into descriptive studies, diagnostic performance assessment, exposure–outcome association analyses, treatment effectiveness evaluation, and prediction modeling. [33, 34]

Table 2 maps selected examples of our previously presented research—shown in italics—onto this framework.

### Diagnostic questions

Within the diagnostic domain, our nationwide survey of anti–PLA2R antibody testing for pMN represents descriptive research. The association between the frequency of MBD marker testing and achievement of guideline-recommended targets was evaluated using an analytic exposure–outcome framework, rather than a classic diagnostic accuracy design. In contrast, investigating the prognostic significance of urinary C-megalin for microalbuminuria onset required an analytic framework designed to assess incremental predictive value beyond.

### Treatment questions

In the treatment domain, our survey of rituximab use for pMN was conducted with a descriptive objective. The evaluation of cinacalcet effectiveness in the MBD-5D Study was designed to address questions complementary to those posed by the EVOLVE trial, using an observational framework.

### Prognostic questions

The study examining the association between long-term dialysis duration and frailty exemplifies an analytic study focused on exposure–outcome relationships. More broadly, when the incidence of a clinically important outcome remains unclear, descriptive prognostic studies may also provide valuable evidence.

A representative example is the timing of vascular access creation in advanced CKD. While the Japanese Society for Dialysis Therapy guideline recommends vascular access creation at an estimated glomerular filtration rate ≤ 15 mL/min/1.73 m^2^, [35] international guidelines often recommend access creation beginning in CKD stage 4. However, studies conducted outside Japan have reported that more than 20% of patients undergoing earlier access creation experience access failure or death before dialysis initiation. We examined the incidence of death or access failure during pre-dialysis CKD stage 5. Among these patients, 89.3% initiated dialysis within 1 year, while only 4.8% experienced death or access failure before commencement. [36] This multicenter descriptive study of approximately 300 patients, led by Dr. Masahito Miyamoto, provided clinically valuable prognostic data and has since been cited in UpToDate and international clinical guidelines. [37]

Finally, an important prognostic question for patients, families, and clinicians is how long patients are likely to survive after initiating hemodialysis. Addressing this requires clinical prediction modeling rather than conventional exposure–outcome analysis. In a project led by Dr. Toshiki Doi, we developed the REC^2^AP score to predict 1-year mortality after hemodialysis initiation. [38] This tool is based on six variables: **R**enal function (estimated glomerular filtration rate), use of **E**rythropoiesis-stimulating agents, **C**harlson Comorbidity Index, serum **C**alcium, serum **A**lbumin, and **P**erformance Status. Scores ranged from 0 to 12, corresponding to predicted 1-year mortality risks from 2.5 to 28.9%. This work highlights the need for specialized approaches to regression modeling and rigorous validation techniques.

### Etiological questions

Clinical research is also essential for elucidating the causes of specific diseases, conditions, or health behaviors. Our study on medication adherence difficulties among patients receiving hemodialysis represents etiological research, identifying health literacy and trust in physicians as important cognitive and relational determinants of adherence.

## Sharpening the lens: the role of the nephrologist–epidemiologist in an era of methodological diversification

I have been fortunate to work at the intersection of clinical nephrology and epidemiology, bridging the gap between evidence and practice through a nephrologist–epidemiologist’s lens. Clinical training allowed me to generate questions grounded in patient care, while epidemiology training provided the tools to address them through appropriate study design and analysis.

Contemporary clinical research now extends well beyond traditional approaches. Mixed-methods research integrating qualitative and quantitative data allows exploration of patient perspectives that cannot be fully captured by numerical measures alone. Deep learning applied to unstructured data, such as medical images, can reveal features undetectable by the conventional screening methods. Machine learning-based clustering further allows identification of subgroups in whom treatments may be particularly beneficial—or harmful.

Large language models may further support this evolving landscape by facilitating literature synthesis, organizing complex information, and assisting clinical reasoning and SDM [39]. However, their uncritical use warrants caution, as such models may confidently generate factually incorrect or contextually inappropriate information (i.e., hallucinations) and may amplify structural biases embedded in training data [39, 40].

Even in the United States, former American Society of Nephrology president Dr. William G. Couser has noted the growing difficulty of excelling simultaneously in clinical care, research, and education. [41] As methodological approaches continue to diversify, mastering them all within a single researcher or a department is no longer realistic. Moreover, many technical aspects of clinical statistics once taught in schools of public health can now be largely acquired through self-directed learning, increasingly supported by large language model tools.

In this context of methodological diversification, time constraints, and emerging benefits and risks associated with artificial intelligence, the one thing a nephrologist–epidemiologist can do is to cultivate a sharpened lens: the ability to discern clinically meaningful questions and to refine them into answerable research questions. However, explicit guidance on how to generate and iteratively refine such clinical questions remains limited. Drawing on my own experience, as well as insights from scientific meetings and previous publications, I summarize practical heuristics for generating and sharpening clinical questions in Table 3. [34, 42]

**Table 3:** Practical tips for conceiving and sharpening clinical questions (CQs)

Situation	Action	Rationale
1. When it is unclear which research topic to pursue	Focus on distinctive clinical practices or patient populations at your own institution	This allows formulation of clinical questions that may not be addressable in other settings
2. When unfamiliar with a topic of interest	Before conducting an unsystematic literature search, carefully read recent high-quality review articles related to the topic	This helps identify unresolved or controversial clinical questions at the current state of knowledge
3. When deriving research questions from clinical experience	• Repeatedly observe the co-occurrence or sequence of two clinical events • Pay attention to irregular or unexpected events	These observations may generate hypotheses regarding potential causal relationships or prompt alternative hypotheses that cannot be explained by existing theories
4. When comparing personal clinical experience with existing evidence	Critically compare observations from routine clinical practice with published data or guideline recommendations and assess the degree of concordance	Discrepancies may indicate clinically important knowledge gaps worthy of investigation

Adapted from a Japanese-language review article authored by the present author. [34]

Looking ahead, this lens can be applied across emerging research contexts, including the use of large-scale real-world data to evaluate infrequent but clinically important outcomes [43], as well as the integration of more granular, PRO data to capture everyday treatment experiences [44]. By nurturing networks of research and education with both current and future colleagues, I hope to pursue clinical research that addresses questions and ultimately contributes to better kidney care.

## Data Availability

Not applicable.
